# Supplementary material for: Scoping Review of Intervention Strategies for Improving Coverage and Uptake of Maternal Nutrition Services in Southeast Asia
Source: Int J Environ Res Public Health. 2021 Dec 16;18(24):13292. doi: 10.3390/ijerph182413292 (PMC8701361; doi:10.3390/ijerph182413292)
Supplement: Supplementary file 1 [file ijerph-18-13292-s001.zip › ijerph-1439525-supplementary.pdf]

Figure S1. Nutrition search strategy in PubMed:

((((((((Evaluate) OR evaluating) OR evaluation) OR effectiveness) OR impact)) AND ((((((((((((((Nutrition program) OR Nutritional Program) OR Nutrition service package) OR Nutrition supplementation) OR Nutrition education) OR Nutrition counseling) OR dietary supplementation) OR dietary education) OR dietary counselling) OR nutrition communication) OR micro nutrients) OR micro-nutrients) OR nutrition intervention) OR nutrition screening)) AND ((((((((((Pregnant) OR Antenal) OR Prenatal) OR gestation) OR conception) OR perinatal) OR intranatal) OR postnatal)) OR pregnancy)) AND ((((((((((((((Maternal mortality) OR maternal morbidity) OR still birth) OR premature birth) OR preterm birth) OR small for gestational age) OR SGA) OR Low birth weight) OR LBW) OR still birth) OR Intra uterine growth retardation) OR IUGR) OR Maternal death) OR neonatal mortality) AND ("last 10 years"[PDat] AND Humans[Mesh])

Table S1. Quality Appraisal Checklist for Randomized Controlled Trials:*

	Bhutta (2009)[12]	Sunawang (2009)[13]	Gernand (2015)[19]	Sablok (2015)[21]	Mridha (2015)[25]	Harris-Fry(2018)[32]	Dewey (2017)[30]	Saville (2018)[35]	Stevens (2018)[38]	Svefors (2018)[39]	Nair (2017)[31]	Nguyen (2017)[28]	Dhaded (2020)
Was true randomization used for assignment of participants to treatment groups?	Yes	Yes	Yes	Yes	Yes	Yes	Yes	Yes	Yes	Yes	Yes	Yes	Yes
Was allocation to treatment groups concealed?	Yes	No	No	No	Yes	Unclear	Unclear	No	No	NA	NA	No	Yes
Were treatment groups similar at the baseline?	Yes	No	Yes	Yes	Yes	Yes	Yes	Yes	Yes	NA	Yes	Yes	Yes
Were participants blind to treatment assignment?	Yes	Yes	Yes	Unclear	Yes	Yes	Yes	No	No	NA	Yes	No	Unclear
Were those delivering treatment blind to treatment assignment?	Yes	Yes	Yes	No	No	No	Yes	No	No	NA	Yes	No	Unclear
Were outcomes assessors blind to treatment assignment?	Yes	Yes	Unclear	No	Yes	No	Yes	No	No	NA	Yes	No	No
Were treatment groups treated identically other than the intervention of interest?	Yes	No	Yes	Unclear	Yes	No	Yes	Yes	Yes	Unclear	Unclear	Yes	Yes
Was follow up complete and if not, were differences between groups in terms of their follow up adequately described and analysed?	Yes	Yes	Yes	Yes	No	Yes	Yes	Yes	Yes	NA	Yes	Yes	Yes

Were participants analysed in the groups to which they were randomized?	Yes	Yes	Yes	Yes	Yes	Yes	Yes	Yes	Yes	Yes	Yes	Yes	Yes
Were outcomes measured in the same way for treatment groups?	Yes	Yes	Yes	Yes	Yes	Yes	Yes	Yes	No	Yes	Yes	Yes	Yes
Were outcomes measured in a reliable way?	Yes	Yes	Yes	Yes	Yes	Yes	Yes	Yes	Yes	Yes	Yes	Yes	Yes
Was appropriate statistical analysis used?	Yes	Yes	Yes	Yes	Yes	Yes	Yes	Yes	Yes	Yes	Yes	Yes	Yes
Was the trial design appropriate, and any deviations from the standard RCT design (individual randomization, parallel groups) accounted for in the conduct and analysis of the trial?	Yes	No	Yes	Yes	No	No	Yes	No	No	NA	No		Yes
Overall appraisal	Include	Include	Include	Include	Include	Include	Include	Include	Include	Include	Include	Include	Include

* *Reference: Tufanaru C, Munn Z, Aromataris E, Campbell J, Hopp L. Chapter 3: Systematic reviews of effectiveness. In: Aromataris E, Munn Z (Editors). Joanna Briggs Institute Reviewer's Manual. The Joanna Briggs Institute. 2017. https://reviewersmanual.joannabriggs.org/. Accessed 18 Jan 2020.*

Table S2. Quality Appraisal Checklist for Analytical Cross-Sectional Studies:*

	Rah (2011)[14]	Nisar (2014)[17]	Jolly (2016)[23]	More (2018)[36]	Stevens (2018)[37]	Wendt (2018)[40]	Kosec (2015)[22]	Nguyen (2017)[27]	Raghunathan (2017)[29]
Were the criteria for inclusion in the sample clearly defined?	Yes	Yes	Yes	Yes	Yes	Yes	No	Yes	Yes
Were the study subjects and the setting described in detail?	Yes	Yes	Yes	Yes	Yes	Yes	Yes	Yes	Yes
Was the exposure measured in a valid and reliable way?	Yes	Yes	Yes	Yes	No	No	Yes	Yes	Yes
Were objective, standard criteria used for measurement of the condition?	Yes	Yes	Yes	Yes	Yes	Yes	Yes	Yes	Yes
Were confounding factors identified?	Yes	Yes	Yes	Yes	No	No	No	Yes	Yes
Were strategies to deal with confounding factors stated?	Yes	Yes	Yes	Yes	NA	No	No	Yes	Yes
Were the outcomes measured in a valid and reliable way?	Yes	Yes	Yes	Yes	No	No	Yes	Yes	Yes

Was appropriate statistical analysis used?	Yes	Yes	Yes	Yes	No	No	Yes	Yes	Yes
Overall appraisal:	Include	Include	Include	Include	Include	Include	Include	Include	Include

**Reference: Moola S, Munn Z, Tufanaru C, Aromataris E, Sears K, Sfetcu R, Currie M, Qureshi R, Mattis P, Lisy K, Mu P-F. Chapter 7: Systematic reviews of etiology and risk . In: Aromataris E, Munn Z (Editors). Joanna Briggs Institute Reviewer's Manual. The Joanna Briggs Institute. 2017. https://reviewersmanual.joannabriggs.org/. Accessed 18 Jan 2020*

Table S3. Quality Appraisal Checklist for Quasi-Experimental Studies:*

	Vir (2014)[18]	Memon (2015)[20]	Rahman (2016)[26]	Pavithra (2019)[41]
Is it clear in the study what is the 'cause' and what is the 'effect' (i.e. there is no confusion about which variable comes first)?	Yes	Yes	Yes	Yes
Were the participants included in any comparisons similar?	Yes	Yes	Yes	Yes
Were the participants included in any comparisons receiving similar treatment/care, other than the exposure or intervention of interest?	Unclear	Unclear	Unclear	No
Was there a control group?	Yes	Yes	Yes	Yes
Were there multiple measurements of the outcome both pre and post the intervention/exposure?	NA	No	Yes	Yes
Was follow up complete and if not, were differences between groups in terms of their follow up adequately described and analysed?	NA	No	Yes	Yes
Were the outcomes of participants included in any comparisons measured in the same way?	Yes	Yes	Yes	Yes
Were outcomes measured in a reliable way?	Yes	Yes	Yes	Yes
Was appropriate statistical analysis used?	Yes	Yes	Yes	Yes
Overall appraisal:	Include	Include	Include	Include

**Reference: Tufanaru C, Munn Z, Aromataris E, Campbell J, Hopp L. Chapter 3: Systematic reviews of effectiveness. In: Aromataris E, Munn Z (Editors). Joanna Briggs Institute Reviewer's Manual. The Joanna Briggs Institute. 2017. https://reviewersmanual.joannabriggs.org/. Accessed 18 Jan 2020.*

Table S4. Quality Appraisal Checklist for Qualitative Research:*

	Kadiyala (2016)[24]	Hashmi (2018)[33]	Ramakrishnan (2012)[15]	Noznesky (2012)[16]
Is there congruity between the stated philosophical perspective and the research methodology?	Yes	Yes	Yes	Yes
Is there congruity between the research methodology and the research question or objectives?	Yes	Yes	Yes	Yes
Is there congruity between the research methodology and the methods used to collect data?	Yes	Yes	Yes	Yes
Is there congruity between the research methodology and the representation and analysis of data?	Yes	Yes	Yes	Yes
Is there congruity between the research methodology and the interpretation of results?	Yes	Yes	Yes	No
Is there a statement locating the researcher culturally or theoretically?	No	No	No	No
Is the influence of the researcher on the research, and vice- versa, addressed?	No	No	No	Yes

Are participants, and their voices, adequately represented?	Yes	Yes	Yes	Yes
Is the research ethical according to current criteria or, for recent studies, and is there evidence of ethical approval by an appropriate body?	Yes	Yes	Yes	NA
Do the conclusions drawn in the research report flow from the analysis, or interpretation, of the data?	Yes	Yes	Yes	Yes
Overall appraisal	Include	Include	Include	Include

**Reference: Lockwood C, Munn Z, Porritt K. Qualitative research synthesis: methodological guidance for systematic reviewers utilizing meta-aggregation. Int J Evid Based Healthc. 2015;13(3):179–187*

Table S5. Quality Appraisal Checklist for Cohort Studies:*

	Khanam (2018)[34]
Were the two groups similar and recruited from the same population?	Yes
Were the exposures measured similarly to assign people to both exposed and unexposed groups?	Yes
Was the exposure measured in a valid and reliable way?	Yes
Were confounding factors identified?	Yes
Were strategies to deal with confounding factors stated?	Yes
Were the groups/participants free of the outcome at the start of the study (or at the moment of exposure)?	Yes
Were the outcomes measured in a valid and reliable way?	Yes
Was the follow up time reported and sufficient to be long enough for outcomes to occur?	Yes
Was follow up complete, and if not, were the reasons to loss to follow up described and explored?	No
Were strategies to address incomplete follow up utilized?	Yes
Was appropriate statistical analysis used?	Yes
Overall appraisal	Include

**Reference: Moola S, Munn Z, Tufanaru C, Aromataris E, Sears K, Sfetcu R, Currie M, Qureshi R, Mattis P, Lisy K, Mu P-F. Chapter 7: Systematic reviews of etiology and risk . In: Aromataris E, Munn Z (Editors). Joanna Briggs Institute Reviewer's Manual. The Joanna Briggs Institute. 2017. https://reviewersmanual.joannabriggs.org/. Accessed 18 Jan 2020.*

Table S6. Characteristics of the included studies (n=31)

Study	Study design	Objectives	Population characteristics	Settings
Bhutta (2009)[12]	Cluster RCT	To evaluate the efficacy and acceptability of the UNICEF maternal multiple micronutrient supplement and its impact on maternal health and birth outcomes	Pregnant women<br>Intervention: Multiple micronutrients (*n* = 1148)<br>Control: IFA supplementation (*n* = 1230)	Urban population (Bilal, Karachi) and rural villages (Kot Diji district, rural Sindh) in Pakistan
Sunawang (2009)[13]	Cluster RCT	To evaluate the effect of supplementation with the recommended multiple micronutrient supplement in comparison with IFA on birth size, pregnancy outcome, and micronutrient status in women.	Pregnant women<br>Intervention: Multiple micronutrients (n=432)<br>Control: IFA supplementation (n=411)	Indramayu district in the West Java province of Indonesia

Rah (2011)[14]	Cross-sectional assessment at the end of the intervention	Assessment of the MNP program implemented as part of the emergency response to cyclone Sidr.	Lactating mothers, mothers of children 12 to 59 months, and postmenarcheal adolescent girls aged 13 to 17 years<br>Intervention: MNP and food ration (n=746)<br>Control: Only food ration (n=696)	Coastal community Barguna, Bagerhat, Patuakhali, and Pirojpur; Bangladesh
Ramakrishnan (2012)[15]	Mixed method; desk review and qualitative approach.	To identify barriers and opportunities for improving maternal nutrition public health intervention in India.	Program officials, health workers at the central and peripheral level, community leaders and volunteers. Women 18-45 yrs. with child ≤3 yrs. old.	Uttar Pradesh: Meerut, Sitapur, Kaushambi, Jhansi<br>Tamil Nadu: Madurai, Thiruvanamalai, Dindigul, Salem, India
Noznesky (2012)[16]	Secondary data analysis combined with key informants interview (May and August 2010)	To examine existing interventions for reducing maternal undernutrition, identify barriers and opportunities for expanding their coverage and quality.	Interview: n=48 and include policy makers, program managers, service providers.<br>Secondary data source: survey reports and program documents.	New Delhi and Bihar, India
Nisar (2014)[17]	Secondary data analysis of a multistage stratified cluster random sampling.	To investigate the effect of use of IFA supplements during pregnancy on the risk of early neonatal deaths	Singleton live births: data Nepal demographic and health surveys and Pakistan demographic and health surveys (2006 and 2011)	National level survey data. Nepal and Pakistan
Vir (2014)[18]	Quasi-experimental mixed methods.	To study the impact of community-based Mitanin programme on nutritional status in NSI and non-NSI group.	3626 households with children <3 years' old<br>Intervention: NSI with Mitanin (n = 1825)<br>Control: Mitanin alone (n = 1801)	Selected district in Chhattisgarh, India
Gernand (2015)[19]	Double-blind, cluster-RCT	Impact of MMN on key foetal growth factors in the context of a randomized trial of MMN compared to IFA.	Pregnant married women in the reproductive age group<br>Intervention: MMN (n=264)<br>Control: IFA supplement (n=236)	Rural North-Western Bangladesh
Memon (2015)[20]	Exploratory quasi-experimental design	To evaluate the impact of the intervention package on perinatal and neonatal mortality and maternal and new-born care practices	Pregnant women<br>Intervention: Intervention package (n=16802 households, of these, 3200 pregnant women received intervention)<br>Control: Routine health services (n=18659 households)	Gilgit district of Northern Pakistan
Sablok (2015)[21]	RCT	To determine the status of vitamin D in pregnancy and evaluate the effect of supplementation on maternofoetal outcomes.	Primigravida with singleton pregnancy at 14-20 weeks,<br>Intervention: n=120<br>Control: n=60	Safdarjung hospital, New Delhi, India

Kosec (2015)[22]	Cross-sectional and secondary data analysis.	To examined the predictors of use of immunization information services, food supplements, pregnancy care information, general nutrition information.	Household and FLW surveys conducted in 2012 in 400 villages in 14 blocks.<br>Survey: 377 AWWs and 382 ASHAs were interviewed.	Bhojpur district in Bihar, India
Jolly (2016)[23]	A Community-based cross-sectional study with controls	Evaluation of maternal health service indicators in urban slum of Bangladesh	1206 married women, aged 15±49 years, with a pregnancy outcome in the previous year<br>Intervention, n=47 slums<br>Control, n=10 slums	Narayanganj City Corporation (IG); Narsingdi Sadar Municipality (CG); Bangladesh
Kadiyala (2016)[24]	A case study of digital green approach using qualitative design	To examine the feasibility of maternal, infant, and young child nutrition BCC through an innovative agricultural extension programme serving nutritionally vulnerable groups.	Women and household members within the 15 intervention pilot village.	Patna and Ghatgaon, two district blocks in the Keonjhar district of Odisha, India
Mridha (2016)[25]	Researcher-blind, longitudinal, cluster-randomized effectiveness trial.	To evaluate the effect of LNS-PLs on birth outcomes.	4011 pregnant women at ≤20 gestational weeks.<br>Intervention: LNS-PLs (n=1047 from 16 clusters)<br>Control: IFA supplements (n=2964 from 48 clusters)	11 rural unions of the Badarganj and Chirirbandar northwest region of Bangladesh
Rahman (2016)[26]	Quasi-experimental study design (5 years)	To evaluate the potential of community mobilization using FLWs for improving health-related outcomes in mothers, neonates, and children less than five years of age	All primi/multi gravid women with youngest child <5 years old<br>Baseline (in hhs):<br>Intervention: 4800; Control: 2400<br>Endline (in hhs):<br>Intervention: 2400; Control: 1200.	Intervention: Nilphamari, Gaibandha, Rangpur, and Mymensingh<br>Control: Naogaon and Netrakona, Bangladesh
Nguyen (2017)[27]	Cross-sectional survey	To examine the maternal, household, and health service factors influencing maternal nutrition practices in the MNCH program areas.	Pregnant women n=600<br>recently delivered women with infants under 6 months of age n=2000	20 rural sub-districts from Mymensingh, Rangpur, Kurigram, and Lalmonirhat, Bangladesh
Nguyen (2017)[28]	Cluster RCT with cross-sectional baseline (2015)/end line (2016) survey	Evaluate the effect of nutrition-focused MNCH compared with standard MNCH on coverage of nutrition interventions, maternal dietary diversity, micronutrient supplement intake, and early breastfeeding practices.	Pregnant women:<br>Intervention: n=300; Control: n=300 recently delivered women with infants under 6 months of age:<br>Intervention: n= 1000;<br>Control: n=1000	20 rural sub-districts from Mymensingh, Rangpur, Kurigram, and Lalmonirhat, Bangladesh

Raghunathan (2017)[29]	Secondary data analysis and household survey.	To assess the effect of Mamata scheme on the exposure to nutrition interventions and house-hold food security.	Mother with children 0-24 months survey data n=1161	Jagatsinghpur, Keonjhar and Kalahandi, India
Dewey (2017)[30]	Researcher-blind, longitudinal, cluster-randomized effectiveness trial.	To evaluate the effectiveness of home fortification approaches for the prevention of maternal and child undernutrition during the first 1000 days.	Pregnant women at ≤20 GA<br>IFA-MNP=1052;<br>IFA-LNS=930;<br>LNS-LNS=1047;<br>Control (IFA)=982	11 rural unions of the Badarganj and Chirirbandar northwest region of Bangladesh
Nair (2017)[31]	A cluster-RCT, 120 clusters, 60 to each group (Oct 1, 2013, and Dec 31, 2015)	To determine whether a strategy involving new worker doing home visit and participatory women group meetings could improve maternal nutrition, feeding, hygiene, care, and stimulation practices for children to increase in children's linear growth.	Pregnant women and their infants:<br>Intervention n=1460<br>Control: n=1541	Singhbhum and Kendujhar, 2 adjoining districts of Jharkhand and Odisha, India
Harris-Fry (2018)[32]	Cluster RCT (Dec 13 to Feb 15)	To measure the effects of pregnancy-focused nutrition interventions on intra household food allocation, dietary adequacy, and maternal nutritional status. (PLA alone, PLA+food transfers, and PLA+cash transfers).	All pregnant women within selected clusters, their mother in law and male households head.<br>PLA=154<br>PLA+cash= 283;<br>PLA+food=218<br>Control=150	Dhanusha and Mahottari districts (in province 2); Nepal
Hashmi (2018)[33]	A convergent parallel mixed-methods design.	To examine perceptions and practices of diet and physical activity among pregnant women in a migrant population along the Myanmar–Thailand border.	Cross sectional survey=388 pregnant women<br>FGD=11 FGD of 66 women<br>IDI= 4 midwives.	2 ANC clinics run by the SMRU in Maw Ker Thai and Wang Pha, Thailand.
Khanam (2018)[34]	Retrospective cohort study design.	To assess the effects of different durations of low-dose calcium supplement during pregnancy on the incidence of PIH.	Pregnant women who gave birth between November 2016 and May 2017, n=11387	10 sub districts of Mymensingh, Rangpur, Lamonirhat & Kurigram; Bangladesh
Saville (2018)[35]	Four-arm cluster-RCT with 20 clusters randomly allocated to each arm.	To compare the effects of three interventions (PLA alone, PLA+food transfers, and PLA+cash transfers) with usual government program on birthweight and child nutritional status.	Married women aged 10-49 years, who had not had tubal ligation and whose husbands had not had vasectomy,<br>PLA plus food, n = 2997;<br>PLA plus cash, n = 3065;<br>PLA alone, n = 2448;<br>Control: n = 2426	Dhanusha and Mahottari districts (in province 2); Nepal

More (2018)[36]	A mixed quasi-experimental and a qualitative study design	To evaluate the community-based management of acute malnutrition to reduce wasting in urban informal settlements of Mumbai.	Pregnant women and children under age 3<br>Intervention area: 150 AWCs<br>Comparison: 107 AWCs in nearby informal settlements of Wadala.	Dharavi, urban informal settlements community in Mumbai, India
Stevens (2018)[37]	A mixed method cross sectional survey and qualitative study design.	To evaluate the impact of a community-based participatory action plan to enhance PFA uptake.	Pregnant women and those within 2 months postpartum seeking care at SMRU clinics<br>PW: baseline =371, endline =307<br>LHW: baseline =100, endline =79	Maela, WangPha and MawkerThai. Thailand-Myanmar
Stevens (2018)[38]	Matched cluster RCT (3rd phase of a multiphased RCT)	To identify whether a locally developed BEP supplementation for undernourished pregnant women affected anthropometric measures at birth and during early infancy.	Undernourished pregnant women with MUAC of ≤22.1 cm.<br>Intervention n=58<br>Control n=29	Pirganj; a sub district of Rangpur, rural areas of Northern Bangladesh.
Svefors (2018)[39]	Factorial randomized trial (Nov 2001 to Feb 2009)	To evaluate the cost effectiveness of the MINIMat interventions of early food and MMS supplements compared with usual timing of food and routine IFA supplement.	Pregnant women spread across 6 intervention groups; EFe60F (*n* =738), EMMS (*n* =740), EFe30F (*n* =739), UFe60F (*n* =738), UMMS (*n* = 740), UFe30F (*n* = 741)	Matlab; a rural sub district, Bangladesh
Wendt (2018)[40]	Cross-sectional and qualitative study (Nov 2011 to July 2012)	To examine the government health system's IFA supply and distribution system and identify bottlenecks contributing to insufficient IFA supply.	IDI with peripheral health workers and program officials n=59<br>Survey: 3 ANMs from each 137 blocks across 8 districts	Eight district in Bihar, India
Pavithra (2019)[41]	A community-based intervention study (December 2012 to October 2014)	To assess the effect of follow up health education on awareness level of mothers, calorie intake, protein intake, and weight gain among 13–60 month-old malnourished children.	Children aged 13-60 months enrolled in the AWCs of RHC Ramanathapuram and PHC Sedarapet.<br>Intervention n=64<br>Control n=64	IG: rural health centre Ramanathapuram, attached to tertiary care institution and a CG: PHC, Sedarapet rural Puducherry, India
Dhaded (2020)[42]	Secondary analysis; (The parent study was an individually randomized, non-masked, multi-site randomized controlled efficacy trial)	To determine the quantitative improvements in the deficits in birth anthropometry resulting from commencing maternal nutrition supplements at least three months prior to conception or in the first trimester of pregnancy	Pregnant Women spread across 3 Arms<br>Arm 1: Received Lipid-based micronutrient at least three months prior to conception (n=1281);<br>Arm 2: Received Lipid-based micronutrient near the end of the first trimester (n=1277)<br>Arm 3: Control (n=1280).<br>Additional protein-energy supplement was given to women whose BMI was <20 kg/m2 for both Arm 1 & 2 till delivery	Resource-poor rural communities in of India and Pakistan.

RCT: Randomized controlled trial, UNICEF: United Nations Children's Fund, IFA: Iron and Folic Acid, NSI: Nutrition Security Innovation, CG: Control Group, MMN: multiple micronutrient, IG: Intervention Group, BCC: Behaviour Change Communication, LNS: lipid-based nutrient supplement, LNS-PL: LNS for pregnant and lactating women, hhs: households, PLA: Participatory Learning and Action, MNP: micronutrient powder, GA: Gestational Age, FGD: Focus Group Discussion, IDI: In-depth Interview, ANC: Antenatal Care, SMRU: Shoklo Malaria Research Unit, PIH: pregnancy-induced hypertension, AWC: Anganwadi centre, PFA: Preconception folic acid, PW: Pregnant Women, LHW: Local Health Worker, BEP: balanced energy protein, MUAC: Mid-upper arm circumference, MINIMat: Maternal and Infant Nutrition Interventions in Matlab, MMS:

multiple micronutrients, ANM: Auxiliary Nurse Midwife, FLW: frontline workers, AWW: Anganwadi worker, ASHA: Accredited Social Health Activist, RHC: Rural Health Centre, PHC: Primary Health Centre, MNCH: maternal, newborn, and child health, EMMS: Early invitation to MMS, UMMS: Usual timing MMS, EFe30F: Early invitation to Fe 30mg and 400 µg folic acid, UFe30F: Usual timing of Fe 30 mg and 400 µg folic acid, UFe60F: Usual timing of Fe 60 mg and 400 µg folic acid, BMI: Body Mass Index

Table S7. Summary of findings

Study	Intervention/Intervention under evaluation	Delivery mechanisms	Findings
Bhutta (2009)[12]	Intervention group: MMN (UNIMMAP)<br>Control group: IFA	FCHW conducted fortnightly house visits and community-based group sessions.	Tablet consumption is 76.7% for IFA and 75.6% for MMN
Sunawang (2009)[13]	Intervention group: MMN (UNICEF/UNU/WHO)<br>Control group: IFA	Daily home visit to dispense the assigned supplements and direct observed consumption.	Increased compliancy of consumption of IFA and multiple micro nutrient Mean intake were 140 and 136 tablets respectively.
Ramakrishnan (2012)[15]	Public health intervention (maternal nutrition programmes India)<br>▪ IFA supplementation.<br>▪ Take-home food rations.<br>Health education and nutrition counselling.	Take home rations delivered through ICDS and other intervention including NEC were delivered through community events and house visits by CHWs, ASHAs.<br>AWWs are responsible for providing ready to eat food supplements on the 15th day of every month to all pregnant women.	Only 27.6% consumed ≥ 100 IFA tablets during the last pregnancy, 4% took deworming medicine during pregnancy, 20.5% received supplementary food and 10.9% received nutrition and health education.<br>Barriers were lack of supply of supplements, quality of food, misconception about intake of certain food products during pregnancy and in case of take home ration; sharing of food with the household.
Noznesky (2012)[16]	Public health intervention in Bihar, India including IFA supplementation, deworming, protein energy supplements and NEC.	Interventions were delivered through the PHC system, the ICDS and PDS.<br>ANMs, ASHAs and male health workers constitute the primary health workforce at the community level.<br>The ICDS delivers a package of health and nutrition services to pregnant at AWC	Only 4% consumed deworming pill during the last pregnancy, 0.6% utilized Anganwadi services.<br>Barriers: resources shortage poverty, lack of awareness, discrimination based on socio-economic status. Policy related barriers such as lack of focus on maternal nutrition, knowledge level of the program implementers about maternal undernutrition and a faulty program management.
Nisar (2014)[17]	Secondary data analysis of IFA supplements in Nepal and Pakistan	Supplement was delivered through static public sector facilities and CHWs program.	Nepal: 70% consumed IFA during pregnancy in the last 5 years, mean IFA intake was 107 (95% CI = 105–109), 58% start IFA ≤ 5th Month<br>Pakistan: 44% consume IFA during pregnancy in the last 5 years, mean IFA intake was 77 (95% CI = 75–79), 31% start IFA at or before the 5th Month
Vir (2014)[18]	Project group (PG): Nutrition Security Innovation (NSI) + Mitanin programme.<br>Control group (CG): Mitanin programme	Intervention was delivered by trained FCHWs known as Mitanins in both the group	80% both groups received IFA with counselling<br>90% in both group received regular supplies of cereals and sugar from PDS<br>IFA tablets intake were higher among the PG<br>46.6% of household in PG planted kitchen garden vs 32.5% in the CG.
Gernand (2015)[19]	Intervention group: MMN UNICEF/UNU/WHO)-Daily<br>Control group: IFA Daily	CHW visit pregnant mother on a weekly basis to provide the supplements for daily consumption from enrolment to 3 months postpartum.	A high compliance to supplements (consumed a median (IQR) of 95.5% (89.1, 98.4) in both the intervention and control groups was observed
Memon (2015)[20]	Intervention area: a. community mobilization and formation of Community Health Committee (CHC) b. Group NEC sessions by trained CHWs	The intervention package was implemented through monthly household visits, one-to-one counselling sessions with	Implementation related outcome not reported

	c. IEC d. Training of lady health worker, CHW and TBA<br>Control area: Received the routine services of governmental and NGOs in the area.	pregnant women and video sessions in communities by LHW and CHW	
Kosec (2015)[22]	ICDS and health/NRHM services delivered through AWWs and ASHAs.	AWWs provide monthly food supplements, nutrition and health education to pregnant women at AWCs and during home visits.<br>ASHAs motivate pregnant women to have institutional deliveries and provide information on nutrition during pregnancy.	About 35% of sample households reported receiving pregnancy care and nutrition information. Pregnancy recorded in the register were more likely to receive pregnancy care information as compare to those whose details was not log in ASHA's register (OR= 2.25, CI = 1.07–4.74; P = .03).
Jolly (2016)[23]	Intervention group: MANOSHI: It is a community-based Maternal, Neonatal, and Child Health (MNCH) care service package.<br>Comparison group: There is no Manoshi package for ANC	CHWs conducted house visit to identify the pregnant women and provided monthly ANC check-ups, calcium tablets, IFA tablets and counselling till delivery.	Higher consumption of IFA in intervention group (65.90% intervention, 46.91% control, P<001) as compared to the control group was observed.
Kadiyala (2016)[24]	The digital green approach to agricultural extension. Participatory production of low-cost videos promoting best practices and broad dissemination through village-level women's self-help groups. 10 videos promoting specific maternal, infant, and young child nutrition practices were produced and disseminated in 30 villages. 10 months.	CHWs mobilized the self-help groups and facilitated PLA. Community members were trained on video production. Dissemination through group discussions with self-help groups. Government FLWs and local people were recruited from villages in the intervention area to 'star' in videos.	Intervention was reported to be well-received by rural communities and perceived as a credible source of information related to health and nutrition and can be an effective additional intervention strategy to existing frontline health services.
Nguyen (2017)[28]	Intensified, nutrition-focused package in addition to the standard MNCH program, the nutrition-focused MNCH additional activities includes: specific diet plan demonstration, free micro-nutrients supplement, weight monitoring, engaging family members, highly trained workers, supervisor and cash incentives for health volunteers based on their performance.	Interpersonal counselling was delivered by Shasthya Kormi (salaried health workers) and a Shasthya Sebika (CHV).<br>In the nutrition-focused MNCH: monthly home visits and 1 on1 ANC sessions for all pregnancy.<br>Community mobilization; husband forums, video shows. Husband of pregnant women were contacted through the forums twice during the pregnancy.	> 96% in IG received IFA and Calcium.<br>40–50% of all mothers reported exposure to video displayed in the community<br>Proportion of IFA and calcium intake, number and quantity of food group consumed and daily intake of macronutrients improved substantially in the IG as compared to the CG
Nguyen (2017)[27]	NEC<br>IFA Supplements<br>Calcium supplements<br>Deworming	FLHWs identified the pregnancies through monthly home visits and provided services. Women received IFA for free from government clinics.	Good nutrition knowledge was associated with higher consumption of IFA (β=32.5, 95% CI:19.5, 45.6) and calcium (β=31.9, 95% CI: 20.9, 43.0)<br>High level of husband's support were more likely to consume IFA (β = 25.0, 95% CI: 18.0, 32.1) and Calcium (β = 26.6, 95% CI: 19.4, 33.7).<br>Early and more PNC visits and free supplements increased consumption rate.<br>Combined exposure to several of these factors was attributed to the consumption of an additional 46 IFA and 53 Calcium tablets.<br>73.3% of pregnant women were visited by CHW.

Nair (2017)[31]	Intervention group: Community based workers known as Su-Poshan Karyakarta were recruited to carry out community interventions through home visits and participatory women's group action<br>Control group: Ekjut coordinators held five participatory meetings with village health sanitation and nutrition committees in between the committees' regular monthly meetings.	Su-Poshan Karyakarta conducted a single home visit to each pregnant woman in the 3rd trimester give counselling on maternal nutrition, facilitates 2-3 participatory meetings with local women's groups in a month.<br>A four-phase participatory learning and action: assessed the health and nutrition situation in their community; decided on actions to take; took action; and evaluate the process.	Study identified five commonly observed problems during pregnancy and food restrictions accounted to about 61% of the maternal related problems.<br>Significant increase in the odds of pregnant women achieving minimum dietary diversity in intervention clusters (aOR 1·40; 95% CI 1·03 to 1·90, p=0·0311).
Raghunathan (2017)[29]	5000 INR (1500; 2nd trimester end, 1500; 3rd month postpartum, 1000; 6th month and 1000 on the 9th month)		Increased likelihood of pregnancy registration and reception of ANC services and IFA tablets form FHWs.<br>Facilitates adherence to intervention<br>The IPW modelling estimates of the ATE shows that CCT was associated with a statistically significant increase in the probability of the pregnancy being registered (1 pp, $p<0.05$), receiving ANC (5 pp, $p<0.01$) and receiving IFA tablets (9 pp, $p<0.01$).
Harris-Fry (2018)[32]	1. PLA: PLA with government-mandated women's groups facilitated by FCHVs<br>2. PLA plus food: PLA plus 10 kg per month of a fortified BEP supplement of wheat-soya blended flour with 10% added sugar called super cereal.<br>3. PLA plus cash: PLA plus NPR 750 (USD7.5) per month, equivalent to the cost of 10 kg of super cereal or two days' wages.<br>4. The control arm: FCHVs delivered usual government outreach services.	FCHV and nutrition mobilizers facilitated the PLA women's group. Strategy implementation included home visits to pregnant women who did not attend groups, showing picture cards in the community, separate meetings with mothers-in-law, adolescent girls, or male family members, rallies on the importance of maternal nutrition, and screening pregnancy-related videos.	PLA with cash or food transfer reported to have higher attendance rate of monthly meeting (80%) as compared to PLA alone. PLA with food transfer significantly improved in the birth weight of the foetus and equity in energy allocation among the pregnant women. PLA with cash transfer improved the dietary diversity and adequacy as well as the supplement consumption.
Khanam (2018)[34]	(i) counselling on the importance of calcium<br>(ii) free delivery of 30–35 calcium tablets (500 mg each) per month until the end of term	SKs and SSs were the groups of CHW implementing the program.<br>SK: Monthly home visit for providing maternal health care services including NEC and distributing of calcium tablets from the first ANC visit while SS: provides essential health care services at the community and facility level	19.8% women consumed < 90 calcium tablets for less than 3 months while 66.0% consumed 90–179 calcium tablets for 3–6 months and 14.2% consumed 180 or more calcium tablets for more than 6 months.<br>Women ≥ 4 ANC check-ups consumed more calcium tablets ($P < 0.001$).
Saville (2018)[35]	1. PLA: PLA with government-mandated women's groups facilitated by FCHVs<br>2. PLA plus food: PLA plus 10 kg per month of a fortified BEP supplement of wheat-soya blended flour with 10% added sugar called super cereal.<br>3. PLA plus cash: PLA plus NPR 750 (USD7.5) per month, equivalent to the cost of 10 kg of super cereal or two days' wages.<br>4. The control Arm: FCHVs delivered usual government outreach services.	FHCVs and nutrition mobilizers facilitated the PLA women's group. Home visits to pregnant women who did not attend groups, showing picture cards in the community, separate meetings with mothers-in-law, adolescent girls, male family members, rallies on the importance of maternal nutrition, and screening pregnancy-related videos	PLA with cash or food transfer reported to have higher attendance rate of monthly meeting (80%) as compared to PLA alone.<br>PLA with cash transfer improved the dietary diversity and adequacy as well as the supplement consumption.

Stevens A (2018)[37]	A community based participatory action plan to increase the uptake of IFA.	Community level campaign, organizing workshops, display of posters and billboards in the health centre, refugee camps and relevant health clinics and distribution of coloured pamphlets. Workshops and group discussion to discuss the issue and plan activities for solution	Improved knowledge of stakeholders regarding NTDs and PFA.<br>Barriers to IFA intake reported in this evaluation were low health literacy, unplanned pregnancy, limited intervention resources, misconception.
Stevens B (2018)[38]	Intervention group: Small business enterprise was established to produce local prenatal food based supplement, BEP supplement (locally available).<br>Comparison group: Received identical services and support except for the supplement	Community nutrition volunteer provided NEC, food based BEP supplement to the pregnant women at the designated centre in the community or home visit, screening for undernutrition through village-level monthly campaigns ANC	Compliance was high among the intervention group; almost all women consumed the full supplement on a daily basis (n = 57).
Wendt (2018)[40]	Universal supplementation of pregnant and lactating women (IFA supply and practices)	ANMs are the primary contact for IFA Supply and distribution. Pregnant women obtain the IFA from district hospital, PHCs, health sub-centre or home visit by ASHA or from AWCs.	Barriers are lack of appropriate IFA need forecasting, delay supplier deliveries<br>Lack of buffer stock Inconsistent training on IFA counselling/distribution.
Dhaded (2020) [42]	Arm 1: Received lipid-based micronutrient at least three months prior to conception;<br>Arm 2: Received lipid-based micronutrient near the end of the first trimester.<br>Arm 3: Control.<br>Additional protein-energy supplement was given to women whose BMI was <20 kg/m2 for both Arm 1 & 2 till delivery.	Home visitor research assistants visited the house in every two weeks to provide the supplements and also to check for compliance. Compliance was assessed by inspection of calendars the women completed daily and by collection of empty, partially eaten, and unused intervention sachets. One sachet daily until delivery.	The mean (± SD) duration of exposure and overall compliance to Supplement 1 in Arm 1 was 72.6 (± 16.8) weeks and 89.6 ± 9.9% respectively, and for Supplement 2 was 26.3 (± 2.2) weeks and 87.7 (± 15.7) %. Supplement 2 was started in 67% of Arm 1 women prior to conception and in an additional 29% during gestation. In Arm 2, 92% of the women received Supplement 2 starting after 12 weeks' gestation. The duration of Supplement 2 was 56.2 ± 25.5 weeks for Arm 1 and 23.7 ± 5.1 weeks for Arm 2.<br>Mean LAZ and WAZ were low with WAZ more impaired than LAZ among the control group as compared to the interventional groups. Eighteen percent of new-borns in control were stunted (LAZ <-2) and 42% were wasted. Twelve percent were preterm deliveries; thirty-four percent had LBW, and 49% of them were SGA.<br>Compliance was calculated for Supplement 1 as the total number of sachets fully eaten divided by the number of days between starting Supplement 1 and delivery. Supplement 2 compliance was calculated similarly; however, the numerator is the total number of Supplement 2 sachets fully or partially eaten.

MMN: multiple micronutrients, UNIMMAP: United Nations International Multiple Micronutrient Preparation, IFA: iron–folic acid, PFA: Preconception folic acid, FCHW: female community health worker, UNICEF: United Nations Children's Fund, UNU: United Nations University, WHO: World Health Organization, CI: Confidence interval, PDS: Public Distribution System, PG: Project Group, CG: Control Group, IQR: Interquartile range, CHC: Community Health Committee, FLW: frontline workers, FLHW: frontline Health workers, NEC: Nutrition, education and counselling , CHW: Community Health workers, TBA: traditional birth attendant, NGO: Nongovernment organization, MNCH: Maternal, Neonatal, and Child Health, ANC: antenatal care, PLA: Participatory learning action, FCHV: Female community health volunteers, BEP: Balanced Energy Protein, NPR: Nepalese rupee, USD: United States Dollar, SKs: Shashthya Kormis, SSs: Shashthya Sebikas, NTD: neural tube defects, ANM: Auxiliary nurse midwifery, PHC: Primary health Centre, ASHA: Accredited Social Health Activist, aOR: adjusted odds ratio, ICDS: Integrated Child Development Services, NRHM: National rural health mission, AWW: Anganwadi worker, AWC: Anganwadi centre, OR: Odds ratio, PNC: postnatal care, CHV: Community health volunteers, IG: Intervention group, INR: Indian rupee, FHW: Female health worker, IPW: inverse-probability weighting, ATE: average treatment effect, pp: percentage point, CCT: Conditional Cash Transfer, IEC: Information Education and Communication; LAZ: length-for-age Z-score; WAZ: weight-for-age Z-score; LBW: low birth weight; SGA: small for gestational age.
